# Acute effects of long‐distance running on mechanical and morphological properties of the human plantar fascia

**DOI:** 10.1111/sms.13690

**Published:** 2020-05-20

**Authors:** Hiroto Shiotani, Tomohiro Mizokuchi, Ryo Yamashita, Munekazu Naito, Yasuo Kawakami

**Affiliations:** ^1^ Graduate School of Sport Sciences Waseda University Saitama Japan; ^2^ Research Fellow of Japan Society for the Promotion of Science Tokyo Japan; ^3^ School of Sport Sciences Waseda University Saitama Japan; ^4^ Department of Anatomy Aichi Medical University Aichi Japan; ^5^ Human Performance Laboratory Organization for University Research Initiative Waseda University Tokyo Japan; ^6^ Faculty of Sport Sciences Waseda University Saitama Japan

**Keywords:** elasticity, mechanical fatigue, medial longitudinal arch of the foot, plantar aponeurosis, stiffness, supersonic shear imaging, thickness, ultrasound shear wave elastography

## Abstract

Long‐distance running (LDR) can induce transient lowering of the foot arch, which may be associated with mechanical fatigue of the plantar fascia (PF). However, this has not been experimentally tested in vivo. The purpose of this study was to test our hypothesis that LDR induces transient and site‐specific changes in PF stiffness and morphology and that those changes are related to the lowering of the foot arch. Ten male recreational long‐distance runners and 10 untrained men were requested to run overground for 10 km. Before and after running, shear wave velocity (SWV: an index of soft tissue stiffness) and thickness of PF at three different sites from its proximal to distal end were measured using supersonic shear imaging and B‐mode ultrasonography. Foot dimensions including the navicular height were measured using a three‐dimensional foot scanner. SWV at the proximal site of PF and navicular height was significantly decreased in both groups after running, with a higher degree in untrained men (−21.9% and −14.1%, respectively) than in runners (−4.0% and −6.3%, respectively). The relative change (%Δ) in SWV was positively correlated with %Δnavicular height in both groups (*r* = .69 and *r* = .65, respectively). Multiple regression analysis revealed that %ΔSWV at the proximal site solely explained 72.7% of the total variance in %Δnavicular height. It is concluded that LDR induces transient and site‐specific decreases in PF stiffness. These results suggest that the majority of running‐induced lowering of the foot arch is attributable to the reduction of PF stiffness at the proximal site.

## 1 INTRODUCTION

The medial longitudinal arch is a unique structure in the human foot. During weight‐bearing exercises, the foot arch lowers while being stretched out and then recoils as the load is removed. Such a spring‐like property of the foot arch helps to attenuate impact forces and store/release elastic strain energy leading to energy saving during running.^1^ It is known, however, that long‐distance running (LDR) imposes repetitive mechanical stress on the foot, thereby inducing transient lowering of the foot arch.^2^, ^3^ As the foot arch is temporarily collapsed, its force attenuation capacity is compromised. A collapsed foot arch (eg, pes planus) is known to increase the risk of injury around the lower limb and foot.^4^, ^5^

Previous work indicates that the foot arch elasticity is primarily attributed to the plantar fascia (PF).^1^, ^6^, ^7^ PF behaves visco‐elastically under tension and contributes to the elastic recoil of the foot arch.^1^, ^8^, ^9^, ^10^, ^11^ During each foot contact of running, PF repetitively experiences tension as high as 0.6‐3.7 times the body weight, with its longitudinal strain up to 6%.^12^, ^13^, ^14^, ^15^ Simulation studies have shown that the tension and peak stress along PF concentrate at its proximal sites.^16^, ^17^, ^18^ Accumulation of such repetitive and site‐specific stress on PF can induce mechanical fatigue (ie, reduction of stiffness and increased strain upon loading).^19^, ^20^, ^21^ This can be a major factor for the lowering of the foot arch during LDR. This potential mechanism should be experimentally tested, but no study has ever attempted to quantitatively evaluate the running‐induced mechanical fatigue of PF in vivo and relate it to the lowering of the foot arch.

Long‐distance runners, regardless of their performance level, are known to be the most prevalent and vulnerable population to plantar fasciitis.^5^, ^22^, ^23^ On the other hand, the plasticity of connective tissues’ mechanical and morphological properties allows them to adapt to chronic mechanical loading (eg, increases in stiffness and cross‐sectional area).^24^, ^25^ Therefore, it is possible that well‐experienced long‐distance runners possess PF and a foot arch that are adapted to LDR (smaller changes in PF properties and arch deformation) as compared to untrained individuals. Available knowledge is limited for this issue. The purpose of the present study was to investigate the acute effects of LDR on the mechanical and morphological properties of PF and the foot arch in untrained individuals and long‐distance runners. The hypotheses were (a) LDR induces transient and site‐specific changes in PF stiffness and morphology, (b) those changes in PF are related to the indices of lowering of the foot arch, and (c) LDR shows smaller changes in PF characteristics and foot dimensions after running in runners than in untrained individuals.

## 2 MATERIALS AND METHODS

### 2.1 Participants

Twenty healthy young men (10 recreational long‐distance runners and 10 untrained individuals; Table 1) participated in this study. All participants had no lower extremity injury in the past 12 months or subjective symptom that would impede running at the time of measurement. The runners had kept habitual running for at least 10 km/wk for the year, and their running experiences ranged between 9 and 16 years. The untrained participants were either sedentary or lightly active, and none of them had been involved in any structured LDR program or continuous sports participation at least 12 months before the measurement. This study was approved by the Institutional Human Research Ethics Committee and was carried out in accordance with the Declaration of Helsinki. Written informed consent was obtained from all participants before data collection.

**Table 1:** Physical characteristics of participants

Variable	Runners	Untrained men	*P *value
n	10	10	‐
Age (y)	22.0 ± 0.7	22.5 ± 1.4	.31
Height (m)	1.68 ± 0.04	1.70 ± 0.05	.39
Body mass (kg)	55.5 ± 4.2	58.4 ± 5.6	.06
BMI (kg/m^2^)	19.6 ± 1.2	20.3 ± 1.7	.11
Running experience (y)	11.0 ± 2.2	‐	‐
Running distance (km/wk)	43.7 ± 35.4	‐	‐
RFS: FFS (n)	7:3	10:0	.06

Note

Data are shown as mean ± SD.

Abbreviations: BMI, body mass index; FFS, forefoot strikers; RFS, rearfoot strikers.

### 2.2 Protocol

Prior to visiting the laboratory, participants were asked not to perform any strenuous exercises for at least 24 hours before the measurements. A test‐retest protocol was used to examine the acute effects of LDR. Participants were requested to run for 10 km on a 700 m outdoor asphalt‐surface circular path adjacent to the laboratory. The protocol was assumed to be identical regarding the mechanical loading for the two groups given that they possess comparable stature (Table 1), and the condition was standardized with the running velocity set at 10 km/h. Lap time was recorded, and running velocity was adjusted by oral instruction. Participants wore their own sports clothes and running shoes during running, but no one used unsuitable shoes that could confound the results (eg, the minimalist, high cushion or high motion control shoes). The foot strike pattern of participants was visually confirmed throughout the running task. All participants were able to complete this 10‐km running task without resting or walking. The average completion time was 0:59:57 (0:59:04‐1:00:57). Before (Pre), immediately after (Post), and 30 and 60 minutes after the termination of running, participants underwent the measurement to examine PF stiffness, thickness, and foot dimensions of their right feet. Care was taken to ensure that participants were in the same posture during pre‐ and post‐running measurements.

### 2.3 Ultrasound measurements

To measure mechanical and morphological properties of PF, supersonic shear imaging (SSI) and B‐mode ultrasonography were used. SSI is a valid and reliable technique to evaluate stiffness and morphology of skeletal muscles, tendons, and fasciae in vivo.^26^, ^27^, ^28^ SSI uses acoustic push pulses that propagate in the soft tissues and measures their velocity (ie, shear wave velocity: SWV) as an index of stiffness.^29^, ^30^ We recently developed a technique using SSI to measure localized SWV values and thickness of PF with high repeatability.^31^

**Figure 1:**
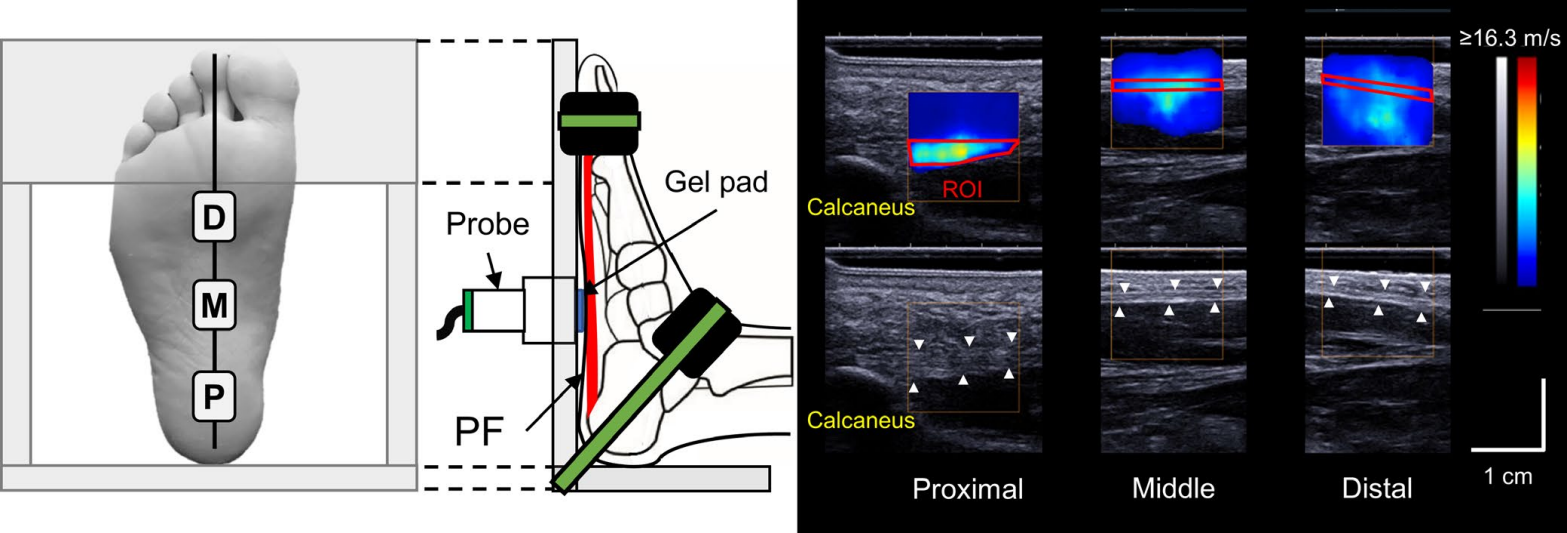
Experimental setup and representative ultrasound B‐mode and shear wave images of the plantar fascia (PF) at the proximal (P), middle (M), and distal (D) sites. ROI, region of interest

Details of measurement and data processing are reported elsewhere^31^; but briefly, B‐mode images and shear wave data were obtained using an Aixplorer ultrasound scanner (version 6.4; Supersonic Imagine) with a linear array transducer (SL 15‐4; Supersonic Imagine). Participants were requested to rest in a supine position on the examination bed with the knee fully extended, and their ankle and toe digits were secured to a custom‐made fixture at the neutral position (Figure 1). PF was scanned in the longitudinal direction at the proximal (in the proximity to the calcaneus), middle (the level of navicular tuberosity), and distal (proximity to the second metatarsal head) sites. The locations of the transducer were marked on the skin surface using a waterproof marker for identical measurement locations. For each measurement site, B‐mode images and shear wave data were recorded for 7 seconds with a system operating at 12 Hz (ie, the default sampling rate of SSI measurement of the current version of the ultrasound scanner). After the data collection, three images separated by 12 frames from the middle of the 7 seconds recording were picked up and used for further analysis.

SWV was obtained as the mean value within the region of interest (ROI) which was manually traced over the fascial boundaries of PF using a measurement tool included in the Aixplorer software (Q‐box^TM^ Trace). Mean ROI size at the proximal, middle, and distal sites were approximately 0.34, 0.22, and 0.17 cm^2^, respectively. Care was taken to exclude any rejection (ie, the area with pixels having no color) within ROI to avoid underestimation of SWV (33 of the 720 images used in this study had rejection areas of approximately 0.01 cm^2^). To assess PF morphology, the distance between the superficial and deep fascial boundaries was measured to determine thickness using a measurement tool (distance). Three images were analyzed for SWV and thickness at each measurement site of PF; then, the three values were averaged to obtain the representative value for each site.

### 2.4 Measurements of foot dimensions

A three‐dimensional foot scanner (JMS‐2100CU; Dream GP) was used to obtain foot dimensions. The scanning and analysis procedure were based on previous studies which reported transient changes in the foot shapes after LDR using the same system.^2^, ^3^ The foot was scanned both in a sitting and standing positions. A laser scanner moved around the foot in an oval trajectory, measuring the foot dimensions and the anatomical marker positions based on laser line triangulation with high accuracy.^2^, ^3^ After the scanning, foot length, dorsal height, and navicular height were obtained. The arch height ratio was calculated as the navicular height normalized to the foot length. Navicular drop was calculated as the difference in the navicular height between sitting and standing positions. Foot dimensions in the standing position are reported as the representative values unless otherwise noted.

### 2.5 Statistical analysis

An unpaired* t* test was performed to test the difference in physical characteristics between groups. The fractions of rearfoot (RFS) and forefoot strikers (FFS) within each group were compared with a Pearson chi‐squared test. Changes in SWV, thickness, and foot dimensions were compared by a two‐way (4 time points x 2 groups) repeated measures analysis of variance (ANOVA). If significant main effects and/or interactions were found, Dunnett's test or an unpaired *t* test was performed as a post‐hoc test, where appropriate. If there was no significant main effect or interactions for ANOVA or significant difference for a post‐hoc test, a post‐hoc power analysis (G*Power v3.1; Heinrich Heine‐Universität) was performed to test our sample size was sufficient for 80% statistical power at a significance level of *α* = .05.

To examine the difference between groups in the degree of running‐induced changes in SWV, thickness, and foot dimensions, relative change (%Δ) from pre‐ to post‐running were calculated for these variables and were compared by an unpaired *t* test between groups. As indices of effect size, partial *η* ^2^ (for ANOVA) and Cohen's *d* (for a post‐hoc test) were calculated. A post‐hoc power analysis estimated that the effect size needed for 80% power was *d* ≥ 0.577. To examine the relationships of individual %Δnavicular height and %Δarch height ratio with %ΔSWV and %Δthickness at each measurement site, Pearson product‐moment correlation coefficients were calculated. Moreover, to determine predictive variables for %Δnavicular height and %Δarch height ratio, six independent variables (%ΔSWV and %Δthickness at each measurement site) with combining data of both groups were entered into a forward stepwise multiple regression model with %Δnavicular height and %Δarch height ratio as the dependent variables. The criteria used for entering and removing the stepwise regression model were *F *≤ 0.05 and *F *≥ 0.10, respectively. Statistical significance was set at *α* = .05. Statistical analysis was performed using SPSS software (SPSS Statistics 25; IBM).

## 3 RESULTS

**Figure 2:**
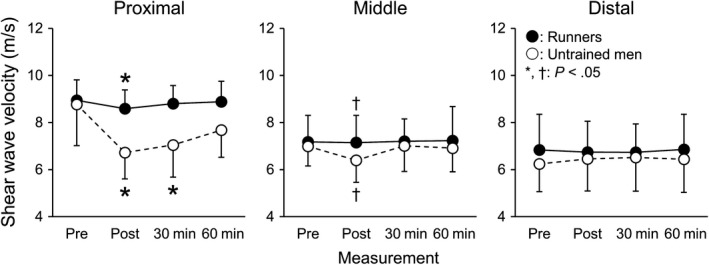
Shear wave velocity of the plantar fascia at the proximal, middle, and distal sites in runners (closed circles) and untrained men (opened circles) measured before (Pre), immediately after (Post), and 30 and 60 min after the termination of running. *Significantly different from pre (*P* < .05). ^†^Combining data of both groups show significant difference from pre (*P* < .05)

Age, height, body mass, BMI, and fractions of the foot strike patterns were not significantly different between runners and untrained men (Table 1). Figure 2 shows the changes in SWV at each measurement site in runners and untrained men. SWV at the proximal site showed a significant time‐group interaction (*P* = .001, *η* ^2^ = 0.374). In runners, SWV at the proximal site significantly decreased at Post (*P* = .045, *d* = 1.026), but not at 30 or 60 minutes after running (*P* ≥ .346, *d* ≤ 0.208). In untrained men, SWV at the proximal site significantly decreased at Post (*P* = .003, *d* = 1.541) and 30 minutes (*P* = .011, *d* = 1.309), but not at 60 minutes after running (*P* = .101, *d* = 0.719). %ΔSWV at the proximal site was significantly smaller in runners than in untrained men (*P* < .001, *d* = 1.912). SWV at the middle site showed a significant main effect of time (*P* = .010, *η* ^2^ = 0.321), without a main effect of group (*P* = .401, *η* ^2^ = 0.040), or their interaction (*P* = .165, *η* ^2^ = 0.266). Dunnett's test with combining data of both groups found that SWV at the middle site significantly decreased at Post (*P* = .036, *d* = 0.643), but not at 30 or 60 minutes after running (*P* ≥ .455, *d* ≤ 0.300).

**Figure 3:**
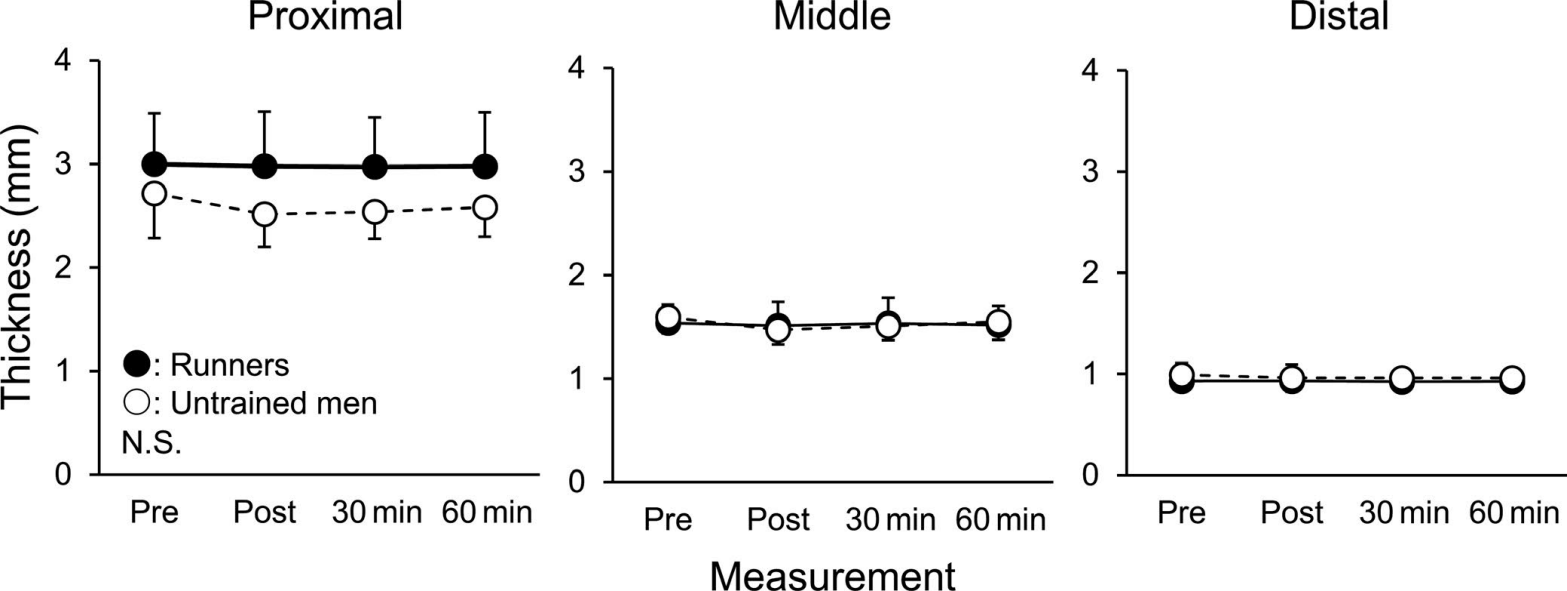
Thickness of the plantar fascia at the proximal, middle, and distal sites in runners (closed circles) and untrained men (opened circles) measured before (Pre), immediately after (Post), and 30 and 60 min after the termination of running

Figure 3 shows the changes in PF thickness at each measurement site in runners and untrained men. PF thickness at the proximal site showed a significant main effect of time (*P* = .012, *η* ^2^ = 0.344) and group (*P* = .048, *η* ^2^ = 0.200), without their interaction (*P* = .072, *η* ^2^ = 0.121). However, Dunnett's test did not find a significant change in PF thickness at the proximal site in runners (*P* ≥ .196, *d* ≤ 0.603) or untrained men (*P* ≥ .196, *d* ≤ 0.603). PF thickness at the middle site showed a significant main effect of time (*P* = .015, *η* ^2^ = 0.174), without a main effect of group (*P* = .947, *η* ^2^ < 0.001) or their interaction (*P* = .115, *η* ^2^ = 0.103). However, Dunnett's test with combining data of both groups did not find a significant change in PF thickness at the middle site (*P* = .211, *d* = 0.429). A post‐hoc power analysis using the parameters of PF thickness revealed that a total of 18 participants (nine participants per each group) were required for 80% statistical power at a significance level of *α* = .05.

Table 2 shows the changes in foot dimensions of runners and untrained men. Navicular height showed a significant time‐group interaction (*P* < .001, *η* ^2^ = 0.342). In runners, navicular height significantly decreased at Post (*P* = .042, *d* = 1.129), but not at 30 or 60 minutes after running (*P* ≥ .576, *d* ≤ 0.163). In untrained men, navicular height significantly decreased at Post (*P* = .036, *d* = 2.029) and 30 minutes (*P* = .043, *d* = 1.506), but not at 60 minutes after running (*P* = .566, *d* = 0.200). Arch height ratio showed a significant time‐group interaction (*P* < .001, *η* ^2^ = 0.329). In runners, arch height ratio significantly decreased at Post (*P* = .044, *d* = 1.050), but not at 30 or 60 minutes after running (*P* ≥ .614, *d* ≤ 0.161). In untrained men, arch height ratio significantly decreased at Post (*P* = .020, *d* = 3.773) and 30 minutes (*P* = .048, *d* = 1.364), but not at 60 minutes after running (*P* = .564, *d* = 0.200). %Δnavicular height (*P* = .002, *d* = 1.655) and %Δarch height ratio (*P* = .001, *d* = 1.679) were significantly smaller in runners than in untrained men. Navicular drop showed a significant main effect of time (*P* < .001, *η* ^2^ = 0.341), without a main effect of group (*P* = .281, *η* ^2^ = 0.064) or their interaction (*P* = .330, *η* ^2^ = 0.061). Dunnett's test with combining data of both groups found that navicular drop significantly increased at Post (*P* = .014, *d* = 0.880) and 30 minutes (*P* = .025, *d* = 0.624), but not at 60 minutes after running (*P* = .586, *d* = 0.742).

**Table 2:** Changes in the foot dimensions in response to long‐distance running

Variable	Runners (n = 10)				Untrained men (n = 10)			
	Pre	Post	30 min	60 min	Pre	Post	30 min	60 min
Foot length (mm)	245.9 ± 8.6	245.7 ± 8.5	244.3 ± 8.2	244.5 ± 7.7	248.3 ± 8.1	248.5 ± 7.8	248.4 ± 7.3	248.2 ± 7.2
Dorsal height (mm)	60.8 ± 4.2	59.6 ± 4.1	60.4 ± 3.9	60.0 ± 4.3	61.1 ± 4.0	60.0 ± 4.6	60.8 ± 4.3	60.8 ± 4.1
Navicular height (mm)^a^, ^b^, ^c^	41.9 ± 6.8	39.4 ± 7.3*, †,‡	40.6 ± 7.4	41.1 ± 7.1	40.9 ± 5.4	35.2 ± 5.6*, †,‡	37.7 ± 5.7*, †,‡	39.8 ± 5.6
Arch height ratio (%) ^a^, ^b^, ^c^	17.1 ± 3.0	16.3 ± 3.1*, †,‡	16.6 ± 3.2	16.8 ± 3.1	16.4 ± 1.9	14.1 ± 2.0*, †,‡	15.2 ± 2.1*, †,‡	16.0 ± 2.1
Navicular height in sitting position (mm)	46.9 ± 5.8	45.5 ± 6.7	46.5 ± 5.7	46.0 ± 6.4	45.9 ± 5.7	42.6 ± 5.5	45.0 ± 5.2	45.4 ± 5.3
Navicular drop (mm)^a^	5.0 ± 2.0	6.1 ± 2.1*, †,‡	5.9 ± 2.8*, †,‡	5.0 ± 2.1	5.1 ± 1.2	7.4 ± 1.5*, †,‡	7.3 ± 2.3*, †,‡	5.6 ± 2.3

Note

Data are shown as mean ± SD.

^a^ Significant main effect of time (*P* < .05).

^b^ Significant main effect of group (*P* < .05).

^c^ Significant time‐group interaction (*P* < .05).

* Significantly different from pre‐running (*P* < .05).

^†,‡^ Effect size is interpreted as large (*d* ≥ 0.8) and medium (0.8 > *d* ≥ 0.5), respectively.

**Figure 4:**
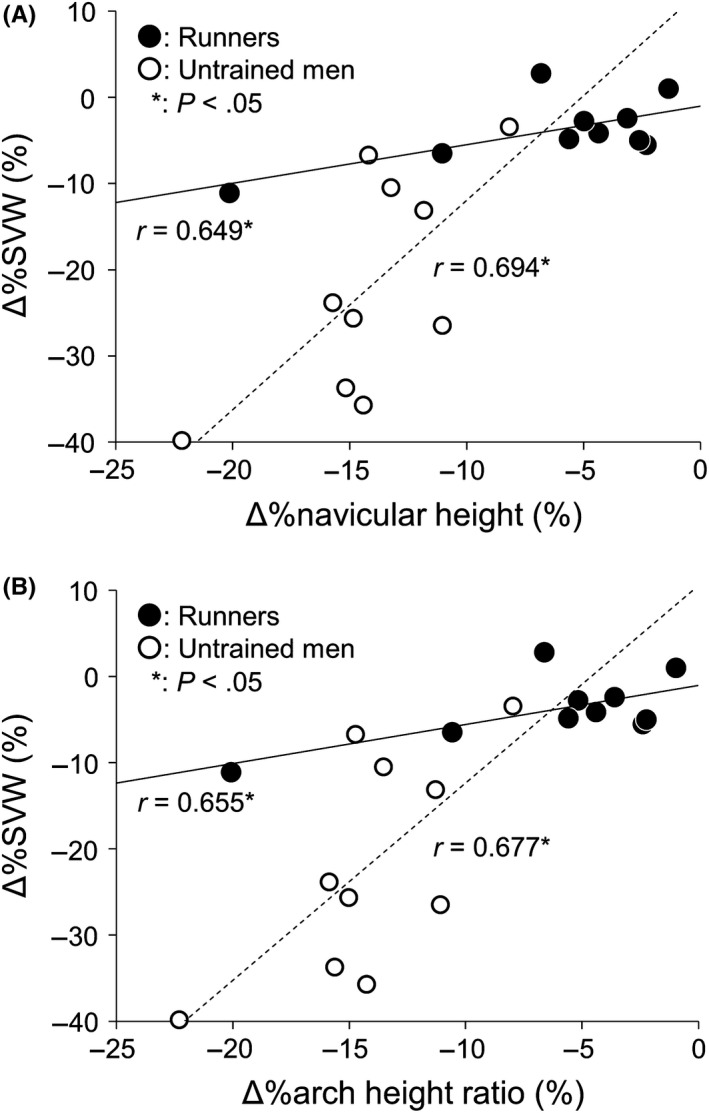
Relationship between the relative change (%Δ) in shear wave velocity (SWV) of the plantar fascia at the proximal site and %Δnavicular height (A) and %Δarch height ratio (B) in runners (closed circle) and untrained men (opened circle). The regression lines are shown with correlation coefficients in runners (bold line) and untrained men (dotted line)

%ΔSWV at the proximal site was positively correlated with %Δnavicular height and %Δarch height ratio in both runners and untrained men (Figure 4). Stepwise multiple regression analysis revealed that %ΔSWV at the proximal site was selected as the single predictor of %Δnavicular height and %Δarch height ratio explaining 72.7% and 74.4% of the variance, respectively.

## 4 DISCUSSION

The most important finding of the present study was that LDR induced transient decreases of both the foot arch and PF stiffness in both runners and untrained individuals and that the two variables were inter‐related. This suggests that mechanical fatigue of PF is one of the causes of foot arch flattening, and in fact, the change in PF stiffness at the proximal site solely explained approximately 70% of the total variance in the measures of lowering of the foot arch. These results support the notion that PF provides a primary supporting base for the foot arch,^1^, ^6^, ^7^ and our study further adds the possibility that mechanical fatigue of PF, in its proximal part in particular, is the key factor for lowering of the foot arch. According to simulation studies, the proximal site of PF is where the mechanical loading is concentrated.^16^, ^17^, ^18^ Such site‐specific stress accumulation during LDR could be the cause of site‐specificity of mechanical fatigue of PF. It may be worthwhile also to note that the proximal site of PF is the common site of plantar fasciitis.^32^ Reduction of PF stiffness can lead to an increase in its strain during running. Mechanical overload and excessive strain can produce microscopic damage within PF which eventually leads to plantar fasciitis,^33^ and our findings are in support of such pathogenesis. Additionally, lowering of the foot arch during running would induce greater eversion of the foot. This can be related to a previous finding that lower extremity joint kinematics in the frontal plane gradually changed (ie, greater eversion of the ankle, greater abduction of the knee, and greater adduction of the hip) throughout a 10‐km running.^34^ Since these kinematic features are known to be the risk factors for the running‐related injuries,^5^ mechanical fatigue of PF and lowering of the foot arch may also increase the injury risk of proximal joints.

Theoretically, repetitive mechanical stress can induce thinning of PF by mechanical fatigue and/or creep deformation.^19^, ^20^, ^21^ Our results did not show this, which was against our hypothesis but it is in line with a previous study on acute effects of walking and running on PF thickness.^35^ A post‐hoc power analysis revealed that our sample size was sufficient to confidently accept or reject our null hypotheses. Our results suggest that PF thickness is unsuitable as an indicator of its mechanical fatigue.

Runners showed smaller changes in PF stiffness and foot arch deformation after LDR than untrained men. This suggests adaptability of PF mechanical properties: runners may have a more resilient PF to protect against the risk of running‐related injury, which was not the case for untrained individuals. However, there was no statistical group difference in the baseline measures. This suggests that the adaptability of PF lies in the way it is under mechanical stress and a potentially faster recovery rate. A previous animal study demonstrated that not only its stiffness but also collagen content, stress‐relaxation, and hysteresis of connective tissues were affected by the external loading during daily exercises.^25^ It is speculated that the parameters such as viscosity, stress‐relaxation, and hysteresis of PF are potential factors for the difference in mechanical fatigue response between runners and untrained individuals. Future studies addressing chronic effects of running on the viscoelastic properties of PF are needed.

**Figure 5:**
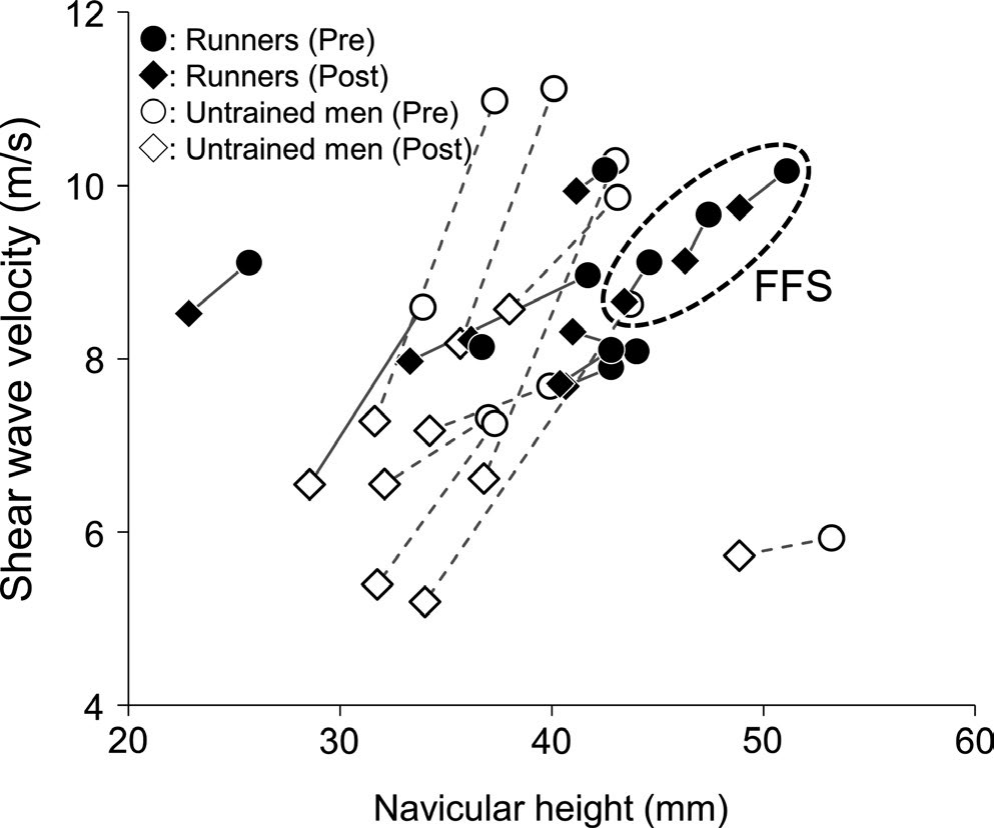
Individual patterns of response in shear wave velocity of the plantar fascia at the proximal site and navicular height in runners and untrained men at pre‐ (closed and opened circle) and post‐running (closed and opened square). Individual changes from pre‐ to post‐running in runners and untrained men are connected with bold and dotted lines, respectively. Runners, forefoot strikers (FFS) in particular, show relatively higher shear wave velocity and navicular height at the baseline

It should be mentioned that smaller change in PF stiffness in runners might also be attributable to the biomechanical differences (eg, kinematics) during running, and indeed, runners showing higher values of SWV and navicular height were FFS while all untrained men were RFS (Figure 5). FFS are considered to receive higher mechanical stress on PF during running with higher velocity,^12^, ^14^, ^15^ which can be one of the reasons for the present results. Chronic effects of running with different patterns on PF properties are unknown at the moment, and further research is warranted to establish the optimal training and conditioning programs that allow injury prevention while being able to run faster.

PF stiffness as well as the foot arch recovered within 60 minutes after running in both runners and untrained men. A previous study on collegiate runners reported that lowering of the foot arch remained for more than a week after a full marathon.^2^ It may be that the persistence of running‐induced fatigue of PF and the arch flattening is related to the duration and intensity of running. As we attempted to set the protocol on overground running, mechanical loads were not measured. This is one of the limitations of the present study. However, it has been shown that there are differences in lower extremity kinematics between overground and treadmill running.^36^, ^37^, ^38^ In addition, treadmill running has negligible effects on the foot arch flattening.^35^, ^39^ Based on these findings, we decided to set the protocol at overground. Previous studies of 10‐km running on a force‐instrumented treadmill at a controlled intensity showed that even in competitive and recreational runners, there were different fatigue responses in kinematics and kinetics.^34^, ^40^ Thus, it seems to be reasonable that runners and untrained individuals showed different fatigue responses in the present results. Since LDR is most often performed overground, we feel that our overground running setting was appropriate to investigate the association of PF and the foot arch in an athletic context. The effect of running duration/intensity on PF and foot arch will lead to a better understanding of running‐induced mechanical fatigue of PF.

In summary, this study revealed that LDR induced transient and site‐specific decreases in PF stiffness, indicating occurrence of mechanical fatigue. Furthermore, the majority of running‐induced lowering of foot arch can be attributed to the reduction of PF stiffness. Long‐distance runners showed smaller changes in PF properties and foot deformation after running compared with untrained individuals. Our results strongly support a current concept that PF is a primary supporting structure of the foot arch, both of which have positive adaptability in response to running training.

## 5 PERSPECTIVES

There are clinical implications for our findings. First, our results highlight that LDR brings about mechanical fatigue primarily in the proximal site of PF. This finding coincides with the pathology of plantar fasciitis. Second, such heterogeneous mechanical properties of the PF depend on running experience. Well‐experienced runners can build up resilient PF that minimize the risk of running‐related injuries. Future studies will enable a better understanding of the optimal training/conditioning schemes that allow PF injury prevention while improving its function as a spring during running.

## CONFLICT OF INTERESTS

No conflict of interest, financial or otherwise, is declared by the authors.
